# Understanding Multilevel Factors Related to Urban Community Trust in Healthcare and Research

**DOI:** 10.3390/ijerph16183280

**Published:** 2019-09-06

**Authors:** Monica Webb Hooper, Charlene Mitchell, Vanessa J. Marshall, Chesley Cheatham, Kristina Austin, Kimberly Sanders, Smitha Krishnamurthi, Lena L. Grafton

**Affiliations:** 1 Case Comprehensive Cancer Center, Case Western Reserve University, Cleveland, OH 44106, USA; 2 University Hospitals Cleveland Medical Center, Seidman Cancer Center, Cleveland, OH 44106, USA; 3 The Gathering Place, Beachwood, OH 44122, USA; 4 Cleveland Clinic Taussig Cancer Institute, Cleveland, OH 44195, USA; 5 NEOMED-CSU Partnership for Urban Health, Cleveland State University, Cleveland, OH 44115, USA

**Keywords:** community-based participatory research (CBPR), community engagement, community listening tour, distrust, healthcare, health disparities, cancer

## Abstract

Background: Community and patient engagement in the healthcare system and biomedical research are prerequisites for eliminating health disparities. We conducted a “listening tour” to enhance our understanding of multilevel factors associated with community trust. Methods: Using community-based participatory research (CBPR) methods, we conducted a phenomenological qualitative study. “Town-hall” style discussions were held at nine sites across an urban, Midwestern city. We recruited adults (N = 130) via community networks, social media, flyers, and word-of-mouth. Demographic assessments were self-administered and listening tour sessions were conducted by trained moderators. Themes were framed within the social ecological model (SEM; intrapersonal, interpersonal, institutional, community, and policy levels). Results: Participants were mostly female (68%), African American (80%), had health coverage (97%) and were diagnosed with a chronic health condition (71%). The overarching theme was sociodemographic differences in distrust, such that African Americans and deaf/hearing impaired participants perceived disparities in healthcare, a lower quality of care, and skepticism about biomedical research, relative to Whites. Conclusions: The depth of distrust for healthcare providers, systems, and researchers in underserved communities remains strong and complex. Findings highlight the need to understand the lived experiences of community members, and how distrust is maintained. Multilevel interventions to increase trust and the accrual of underrepresented populations into clinical trials are needed.

## 1. Introduction

Health disparities are the result of a complex interaction of social, economic, cultural, health system, and political factors. There is a disproportionate and undue burden of chronic medical conditions and death (e.g., cancer [1] diabetes [2], and stroke [3,4]) among low socioeconomic status (SES) and racial/ethnic minority populations. The social ecological model of health (SEM) provides a conceptual framework for understanding the dynamic and interacting factors that underlie health and health behavior(s) [5]. These determinants of health include intrapersonal (e.g., attitudes, beliefs, and knowledge), interpersonal (e.g., family, patient-provider relationships), organizational (e.g., healthcare systems), community (e.g., built environment), and public policy (e.g., resource allocation, health insurance access), each of which contribute to outcomes [5]. The SEM represents a holistic view of the interrelationships among upstream and downstream factors that influence opportunities for optimal health. In underserved populations, in particular, the influence of upstream structural factors may negatively affect individual-level potential for adaptive health behavior engagement. As such, achieving the goals of health disparity reduction and elimination will require a major collective effort (e.g., legislators, health systems, medical providers, scientists, community stakeholders, families, and patients).

Community and patient engagement are prerequisites for positive inroads toward reducing and ultimately eliminating health disparities. The Case Comprehensive Cancer Center (Case CCC) Community Advisory Board (CAB), which represents patient and community voices, identified low levels of trust in healthcare and biomedical research as important factors to understand and address. Thus, the overarching goal of this CBPR [6] effort was to deepen our understanding of multilevel factors related to community trust in an urban, Midwestern city. Consistent with the principles of CBPR, this study was an academic-community partnership, during which the researchers and the CAB each contributed their expertise, experience, and time at each stage of the research process [6]. 

Previous research has examined levels of trust in the healthcare system and research, although much remains to be learned in this domain. The literature indicates that racial/ethnic minorities have significant distrust overall, and in comparison to Whites [7,8]. Byrd et al. found that lack of trust was a primary reason for unwillingness to participate in biomedical research among intergenerational African American males [9]. Even after controlling for SES sex, age, and risk for cardiovascular disease, Braunstein et al. [8] found that African Americans were less willing to participate in pharmacology trials. Racial differences in medical research distrust and harm-related perceptions partially mediated this relationship. A recent qualitative study focused on the influenza vaccine found deep distrust in pharmaceutical companies due to perceived profit motivation [10]. Racial differences in trust for federal institutions was a major theme, such that African Americans described greater distrust compared to Whites [10].

Medical distrust is important to understand and address, as it may also contribute to poor patient outcomes [11], low healthcare quality [12], and increased healthcare costs [13]. Such experiences and subsequent distrust is also related to the underrepresentation of various minority groups in cancer and other clinical trials [9]. In sum, the combination of medical and research exploitation and abuse experienced by disenfranchised populations, perceived discriminatory experiences, and perceived lack of community benefit [14] have led to greater medical distrust among African Americans compared to Whites [15,16,17,18]. Biomedical researchers seeking to recruit racial minorities for clinical trials have also expressed concern about the challenges of engagement due to negative beliefs [7]. With the current national focus on obtaining diverse samples for precision medicine initiatives (e.g., the National Institutes of Health’s All of Us effort), research is needed to better understand the nuances of medical and research distrust in underrepresented and underserved populations, and steps to address this issue must be undertaken.

### The Present Study

The purpose of this study was to enhance our understanding of social contextual factors that influence community trust for healthcare systems and research. Applying the SEM as a conceptual framework, we focused on the lived experiences of residents, with attention to policy, community, institutional, interpersonal, and intrapersonal issues that affect trust levels. We sought to recruit a sample diverse in race/ethnicity, SES, and health and disability status—and engage them in an open-ended forum for sharing thoughts, ideas, and concerns about cancer prevention and control, the healthcare system, and academic/research institutions. Among the goals of the study was to identify disparities in healthcare and/or research experiences that may elucidate distrust levels.

We utilized a phenomenological listening tour approach, which in contrast to focus groups, allowed organic discussions and concentrated on the meanings of participant experiences. Our qualitative approach facilitated a deep assessment of a range of factors related to community trust. Finally, this study was unique from previous research, due to its simultaneous focus on both healthcare and research distrust, allowing us to understand how one informs the other.

## 2. Materials and Methods

### 2.1. Community Advisory Board

The CAB was established in 2012, and its overarching goal is to advise on catchment area, health disparities, and/or health equity research efforts within the Case CCC. The vision of the CAB is to maintain a partnership with Case CCC leadership, researchers, clinicians, and outreach teams to have a positive impact on local cancer care and outcomes, and to represent the voices of the community. The need to better understand community trust for healthcare and research was a CAB-identified priority. Thus, we undertook this initiative as an academic-community research team, guided by principles of CBPR, with a commitment to a systematic approach to understand community resident experiences and perspectives, and to disseminate the findings to the community [19].

### 2.2. Study Design

The Forward Movement Project (Phase I) (see Supplementary Material) was a qualitative study to identify factors related to community trust for local healthcare systems, perceptions about biomedical research participation, and cancer-related experiences by learning from residents and other key stakeholders. We implemented a community “listening tour” approach in nine neighborhoods in an urban city in the Midwest (United States).

### 2.3. Participants

Participants were recruited from the community using several approaches. We utilized the community contacts of the CAB to convene groups for the listening tour sessions. We partnered with a city councilman and community organizations, who distributed flyers to their constituents on behalf of the project. We also recruited participants via social media, email listservs, and widespread flyer distribution. Inclusion criteria required participants to be ≥18 years old and reside within the 15-county Case CCC catchment area.

### 2.4. Procedures

This study was approved by the Case Western Reserve University Institutional Review Board (IRB). The Community Trust and Engagement Working Group, a subcommittee of the CAB, worked closely with the Office of Cancer Disparities Research to develop and implement the study plan. We developed two instruments for this study, which reflected the overarching goal of the project—to allow community voices to be heard with very few questions from the moderator. We enlisted the partnership of key community stakeholders to assist with the identification of locations to conduct listening tour sessions. We identified sites (e.g., community centers, grassroots organizations, barber shops, and health centers) in nine neighborhoods with the capacity to seat 10–50 individuals. Next, we implemented our recruitment plan (described above). Interested community residents contacted the project’s telephone number to register for an upcoming session or enrolled on site.

Listening tour sessions were led by a trained moderator, who guided participants through the informed consent process and completion of a brief demographic assessment. Listening tour sessions were audiotaped and transcribed verbatim. To facilitate anonymity, participants were assigned identification numbers, which they stated prior to commenting or responding to questions. The 90–120 min sessions were designed to be participant-driven. The moderator offered the initial question, encouraged proactive participation among residents, and allowed conversation to ensue naturally. The moderator introduced new questions once no new ideas were offered. Meals and refreshments were provided at each listening tour session, and participants received a $10 gift card for their participation. After data analysis, we developed an infographic summarizing the main findings, which was distributed throughout the community (i.e., at each listening tour site, to community partners, and via email and social media).

### 2.5. Measures

#### 2.5.1. Demographics

Participants self-reported age, sex, annual income, race/ethnicity, and chronic disease presence, such as cancer, diabetes, or high blood pressure (yes/no).

#### 2.5.2. Moderator Guide

The moderator guide consisted of 11 standardized open-ended questions. Questions assessed (a) experiences with major local healthcare systems, (b) level of community trust for local healthcare systems, (c) willingness to participate in biomedical research, (d) views on cancer prevention, and (e) expectations for provider/patient relationships. Questions included: (1) Talk about your experiences with the healthcare system. What instances stand out in your mind and why? (2) Do you trust the local hospitals to provide you with effective treatment and care? (Why or why not?) (3) What things get in the way of you getting the healthcare you need? (4) What are your thoughts about ways to remove or reduce those barriers? (5) Lots of research is going on at the university and hospitals. What are your thoughts on research? Would you participate in studies and/or clinical trials? (Why or why not?) Do you know anyone who has? (6) What can healthcare providers and researchers do to gain and maintain your trust? (7) There are high rates of certain cancers in our local community, such as lung cancer, breast cancer, and prostate cancer—and some groups have higher rates than others (African Americans versus Whites). Talk about this problem. What are the factors that add to these high rates? What can be done to reduce cancer in our communities? (8) Discuss your views on each of the major healthcare systems in our local community. (9) Talk about how connected you think our community is to the healthcare systems. (10) What kind of relationship would you like to have with your healthcare provider? Describe the desired relationship with your provider.

### 2.6. Data Analyses

Descriptive statistics, including frequencies and means (standard deviations), were conducted to summarize sample characteristics. NVivo 11 was used to develop codes, and transcriptions were double coded by a second team member, who was trained by the Principal Investigator. Qualitative data were analyzed using phenomenological content analysis [20], which describes the lived experiences of participants, in the context of the five-stage framework [21]. In stage 1, transcriptions of each listening tour session were verified for completeness and accuracy. In stage 2, the data were indexed into nodes and thematic chunks. A coding manual was used to identify themes and sub-themes related to community trust for healthcare and research. In stage 3, two independent team members coded each transcription by applying standardized codes for themes and sub-themes. The codes were compared for reliability, and any discrepancies (these were minimal) were reviewed and resolved. We converged the data into charts containing the statements/comments/experiences shared within each thematic area. In stage 5, themes were clarified and mapped on to the levels of the SEM framework.

## 3. Results

### 3.1. Participant Characteristics and Overall Themes

We enrolled 130 participants across the listening tour stops. Participants self-identified as African American (80%), non-Hispanic White (17%), or “other” (3%). They were mostly older (between 60–80 years of age), female (68%), had health insurance (97%), diagnosed with a chronic health condition (71%); 10% were deaf/hearing impaired.

Overall, we identified a theme of low trust in the healthcare system due to current and past experiences. Findings also revealed disparities by race/ethnicity and disability status. Positive healthcare experiences were described primarily by non-Hispanic White community members—negative encounters were expressed overwhelmingly by African Americans and those who were deaf/hearing impaired. There were many examples of the complexity of healthcare and research distrust in underserved populations. Themes revealed poignant differences in lived experiences among African Americans and deaf/hearing impaired participants, which impacted attitudes, beliefs, and trust for healthcare institutions and biomedical research. Compared to non-Hispanic White participants, the expressed attitudes, concerns, and barriers to optimal health of the underserved participants in this study revealed intrapersonal level barriers to trusting relationships with healthcare systems, medical providers, and researchers, such as concerns about the true goals of hospitals (i.e., making money over patient care), beliefs that doctors receive compensation for prescribing certain medications, and concerns about biomedical research and participant exploitation. Table 1, Table 2, Table 3, Table 4 and Table 5 provide illustrative examples of narratives associated with distrust in the context of the SEM framework.

### 3.2. Intrapersonal

Themes at the intrapersonal level revealed dramatic differences in lived experiences and consequent beliefs among participants, which impacted patient treatment-decisions, health behavior, and trust for biomedical research. An overall theme of deep distrust emerged, reflected by individual characteristics such as beliefs and attitudes, perceived discrimination due to race/ethnicity or economic status, and personal experiences. As shown in Table 1, patients described negative experiences with healthcare systems and medical providers, which reduced trust. Participant narratives highlighted deep distrust due to beliefs that they were improperly medicated, intentionally misled, or not informed fully about possible harm. They also perceived substandard treatment due to their racial group or income level, and concerns that they may lack the knowledge to advocate effectively for themselves and their families. Several participants detailed the process of being diagnosed with cancer, noting a lack of physician empathy when informed initially about their condition, and recommendations for unnecessary surgery—prevented only by a second opinion. Thematic analysis also revealed significant distrust related to research, which affected potential participation. Despite recognizing the importance of research for new discoveries, medical advancements, medications, and participation for altruistic purposes, participants expressed distrust for research. As illustrated by the representative statements in Table 1, trust for medical research was damaged significantly in the African American community years ago, and has not been repaired. Many participants expressed concerns about enrolling in research clinical trials testing new medications, although they viewed survey-based studies as acceptable. They discussed the history of racist experiments and current exploitation fears (e.g. being, used as guinea pigs) or that their health might be worsened by harmful drugs or placebo pills. A sub-theme of enhanced distrust emerged due to failure to disseminate findings back to participants and the community.

### 3.3. Interpersonal

Themes at the interpersonal level revealed challenges with patient-provider communication and culturally appropriate care (Table 2). Patients who reported positive experiences described medical providers who spent more time talking and listening to them. In contrast, many participants detailed adverse experiences with providers or medical teams. Themes also revealed significant sociodemographic differences in interpersonal patient-reported experiences that affected trust. Specifically, levels of distrust were markedly higher among African Americans and hearing/impaired participants, who were skeptical of provider intentions, behavior, and treatment decisions. They expressed concern about providers giving them incorrect information, guessing versus diagnosing the problem, and over-prescribing medications for “kick-backs” from the pharmaceutical industry; they also emphasized the need to hold doctors accountable. They described feeling rushed due to the provider’s need to see as many patients as possible, poor verbal and non-verbal communication including the use of medical (versus lay) terminology, limited eye contact, and a lack of empathy. A subtheme emerged regarding perceived discrimination and biases during clinical encounters. Some African American participants stated that providers had not “touched” them during their visit, which was perceived as attributable to their race/ethnicity. Deaf/hearing impaired participants described cultural incompetence among medical teams, such as failing to direct communications to them (as the patient), instead turning to their family members—including minor children—as if they were not present. Underserved participants indicated a preference and greater trust in culturally matched or experienced providers, which they stated would increase trust.

### 3.4. Institutional

Themes at the institutional level revealed challenges with healthcare as a system. The overarching theme was that healthcare is “big business” and that revenue generation, as compared to patient care, is of paramount importance. Challenges with access to the health systems, such as difficulty securing timely appointments, the lack of availability of evening and weekend appointments, and difficulty parking and locating specific clinics within large hospital buildings were identified. The depth of institutional factors associated with healthcare and research distrust was substantially greater among both racial minorities and deaf/hearing impaired participants (Table 3). While there was overall agreement that health systems are businesses, few White participants raised this as a trust-affecting concern. In contrast, content analyses identified several themes of institutional distrust among African Americans. Participants expressed beliefs that doctors, hospitals, and pharmaceutical companies are collaborating to “push” medications, even those of unknown effectiveness or with significant side effect profiles. Participants also perceived that the quality of patient care varies based on insurance type, neighborhood/home address, age, and race/ethnicity, such that those who are insured by Medicaid, live in low-income areas, are older, or are African American receive lower quality care. Aspects of the hospital climate, particularly private hospitals, such as impersonal interactions, long wait-times, and limited discussion of patient support services also increased distrust and the feeling of being unwelcome. Indeed, underserved participants were largely unaware of services such as patient navigation, social work, and financial assistance programs. Our deaf/hearing impaired participants expressed strong dissatisfaction with the use of video remote interpreters (VRI) instead of ensuring that live interpreters are available. Healthcare experiences were also adversely affected by the lack of institutional accommodations for deaf/hearing impaired patients, such as hiring qualified live interpreters, and training for providers and staff on how to use the VRI and cultural humility specific to this community.

### 3.5. Community

We also identified community level themes affecting trust (Table 4). These themes focused on the built environment, businesses, accessibility to healthcare organizations, relationships between healthcare systems and community-based organizations, and environmental injustices that may affect health. Underserved participants, who lived in disadvantaged neighborhoods or who would benefit from disability services, expressed trust-related factors almost exclusively. Specifically, environmental exposures in low-income neighborhoods were perceived to contribute to the high prevalence of health conditions, including cancer and lung disease. They described high levels of air pollution, trash dumping, and contaminated water. Participants also described a lack of access to nutritious foods, living in food deserts, and the prevalence of stores that would be unallowable in suburban communities. Access to healthcare also emerged as a theme, specifically the distance to medical clinics in low-income areas, the inappropriate placement of private hospitals that do not accept Medicaid in poor neighborhoods, and the lack of nearby trauma centers and emergency rooms. Also expressed was the perception that seeking medical treatment in higher-income neighborhoods would result in better healthcare. An additional theme focused on the lack of connection between healthcare systems and communities with high need. Specifically, participants described a desire for health system engagement with grassroots organizations to implement awareness and education programs in comfortable environments. A subtheme focused on the need for healthcare systems to work with community organizations serving the deaf/hearing impaired community for education and training, and to identify culturally appropriate live interpreters.

### 3.6. Policy

The primary themes at the policy level were difficulty managing health insurance and the cost of medical care (Table 5). Most participants had some form of insurance, yet described difficulty understanding covered services, and other specific policy matters. Again, themes demonstrated racial disparities, such that African Americans expressed deep distrust for the policies set by insurance companies and associated governmental laws, whereas most White participants did not raise this concern. African Americans expressed concern about requirements for referrals to see specialists, the financial burden of repeated co-pays, unexplained policy changes that led to their main providers becoming out-of-network, and that certain hospitals did not accept their insurance. Participants were distrustful of high medical bills that arrive months post-visit that require unmanageable out-of-pocket costs, and list services/tests that patients did not recall. Some participants questioned the limited Medicare coverage for treatments and medications. For instance, one participant believed that social security benefit age requirements were based upon the government’s knowledge of the lower life expectancy of some groups (i.e., patients would die before reaching the age to receive benefits to which they are entitled). Underserved deaf/hearing impaired participants talked a great deal about the need for policy interventions to ensure capacity for live interpreters, including guidelines for medical school students in working with this population. Overall, deaf/hearing impaired patients believed that policy change would be necessary to improve healthcare experiences for patients with sensory disabilities. This would include flexible insurance coverage so that patients could select deaf/hearing impaired “friendly” providers to improve the quality of care. Finally, there was a significant theme among both African Americans and deaf/hearing impaired participants regarding the profit motivation of health insurance companies, rather than providing comprehensive healthcare that meets patient needs.

## 4. Discussion

The Case CCC CAB identified community trust as an important concern in the underserved communities surrounding the cancer center. This study applied the SEM framework to identify multilevel factors that affect community residents’ experiences and perceptions around healthcare and research. Overall, themes revealed significant distrust for both healthcare and research, particularly among underserved community members. Intrapersonal themes associated with distrust included individual beliefs and attitudes, perceived discrimination due to race/ethnicity or economic status, concerns about research exploitation, and personal experiences. Interpersonal factors affecting trust reflected adverse experiences in the healthcare setting, concerns about provider behavior, patient-provider communication challenges, perceived discrimination, and lack of cultural humility. Institutional themes related to distrust included views that healthcare organizations are motivated primarily by revenue generation rather than patient care, and inappropriate patient accommodations. We also identified community-level themes such as limited health-related resources, community-to-health system disconnection, and challenges within the built environment. Themes related to policy were also identified, specifically related to the government and healthcare insurance costs, policies, and laws. Underserved patients desired change in the healthcare system and how researchers connect with members of the community.

The observed multilevel racial differences in healthcare distrust found in the current study are consistent with previous research. Guerrero et al. [22] found that African Americans reported substantially lower healthcare trust compared to non-Hispanic Whites, even after adjusting for SES. A recent study found that African Americans expressed greater pharmaceutical company and government distrust than Whites, as these institutions were viewed as profit-motivated and with questionable interests [10]. Deepening our understanding of healthcare distrust is important for addressing health disparities. Trust level and connectedness between community members and healthcare systems may serve as underlying mechanisms for preventive health visits, cancer screening, medication adherence, and health behaviors. Most participants in the current study reported being diagnosed with a chronic medical condition. Previous research suggests that hesitation to access general medical care and/or treatment for chronic conditions is linked closely to healthcare distrust. Webb et al. [23] found that African American adults were less likely to have seen a doctor in the last six months for a medical condition. Due to medical distrust, participants in the current study reported limiting their engagement in healthcare systems unless absolutely needed (e.g., unable to manage symptoms, natural treatments unresponsive, etc.). Thus, understanding individual-level sources of distrust is important for quality improvement initiatives and improving healthcare experiences for groups who experience health disparities.

The current findings were also consistent with previous studies of racial/ethnic differences in distrust for research. The theme of skepticism expressed in the current study was rooted largely in knowledge of past exploitation by researchers and the government, rather than personal negative experiences. African Americans’ distrust for research is longstanding and pervasive, and reasonably so [7]. Our findings extend this work by identifying specific conditions under which participants were amenable to research participation. Participants reported greater distrust for biomedical (e.g., drug) studies relative to survey-based behavioral research. In part, this was due to concerns about exploitation and abuse (e.g., being used as guinea pigs, experimentation without consent), which are fears that have been noted in previous research [7,8]. However, a noteworthy theme across racial/ethnic groups in the current study was recognition of the importance of research and its potential to benefit others. Previous research has reported a similar finding, such that African American males were indeed willing to participate in research, under specific circumstances (such as personal disease status) (5). Consistent with previous research [24], we recommend culturally responsive approaches to increase research participation among racial/ethnic minorities.

Provider-patient challenges at the point of the clinical encounter also emerged as a significant theme associated with distrust. Throughout the listening tour, participants expressed concerns regarding the limited amount of time allocated for appointments, inattentive providers, insufficient treatment explanations, and provider bias due to their race/ethnicity or income. Specific communication challenges were identified among deaf/hearing impaired participants, such as use of the VRI versus live interpreters, lack of visual aids during visits, and communication being directed at significant others instead of the patient. Previous research on patient-provider relationships and underserved populations reported similar themes as they relate to both healthcare [25] and research participation [8]. Systems factors, such as ensuring that patients see the same provider for regular care, have the potential to increase trust [16].

The application of the SEM extends the literature in this domain. This study demonstrates that community distrust is a highly complex problem that transcends individual-level factors. Themes demonstrated that low trust and distrust also result from perceived inequities created and maintained by institutions, community structural factors, and policy. Previous research applied the SEM to address accrual challenges to a genetic-epigenetic clinical trial, and demonstrated success [26]. However, little previous work has applied this framework to understand multilevel factors associated with healthcare and research distrust, identify racial/ethnic differences in these factors, or describe the perspectives of deaf/hearing impaired individuals. Participants identified ongoing concerns in their neighborhoods that increase risks for cardiovascular disease, such as exposure to environmental pollutants [27]—which have implications for trust. The findings from this study can inform institutional efforts to better serve patients and communities, and policy briefs (e.g., city, state, national) to achieve health equity.

A primary strength of this study was the CBPR approach. CBPR allows for innovative academic-research partnerships, explores local knowledge and perceptions, and empowers community stakeholders to act as agents who can investigate community-identified priorities using established research methods [28,29]. Partnering with community organizations increases the credibility of the project, enhancing its usefulness by aligning it with what the community perceives as social and health goals, and provides an avenue for research participants who have varied skills, knowledge, and experiences to contribute to complex problems in complex situations [30]. Our team gathered information highlighting the depth of community distrust in healthcare and research among disadvantaged populations, created a solid base of knowledge from community residents, generated potential avenues to make an impact, and disseminated the findings into the community. Study limitations include the self-selection of participants in one geographic location, and that the age of the sample may not represent the experiences of young adults. Additional information might have been generated by structured interviews; however, our team made the decision to “listen” and resist the desire to collect more than minimal data. Notwithstanding the limitations, this study contributes to the literature on health disparities by using a community engagement approach to inform academic and healthcare systems about the lived experiences of community residents regarding research participation and the quality of healthcare.

## 5. Conclusions

In conclusion, gaining healthcare and research trust in disenfranchised communities represents a marathon rather than a sprint. Our collaborative is committed to empowering communities to achieve optimal health by both helping individuals find their voices and providing tangible recommendations to decision makers within the healthcare system and research enterprises. Future research directions include implementing and testing multilevel strategies to improve patient experiences with the healthcare system and in biomedical research, and evaluating dissemination and implementation science approaches to address health equity. Our ultimate goal is to integrate the knowledge gained in this research to benefit communities.

## Figures and Tables

**Table 1:** Intrapersonal factors that influence trust for the healthcare system and/or research: Illustrative examples.

Social Ecological Model Level	Representative Quotes
Intrapersonal—e.g., individual knowledge, attitudes, behavior, skills, health literacy, financial resources, values, expectations, and sociodemographics	“I think they should give the same healthcare that they would give to their parents or they would like someone to give to them. If you don’t care for your parents, you sure aren’t gonna care for little dark skinned me.” “There is a whole population of my age group who is not showing up, who is not getting this information, who is not going to the doctor, who is not taking their kids to the doctor because of their distrust in the system or they don’t have time. It’s not that we do not want to go to the hospital, but there is a lack of access and a lack of information in our community. There is a lack of just connectedness in our community to pull someone along. I don’t trust the healthcare system at all. I have to be my own best advocate and my family’s advocate. I have to question all the time.” “I think that as a person of color, the most important thing to me was that I felt I was getting the same standard of treatment as everyone else. When I went to go see the first oncologist, my sister went with me and my sister questioned why I can’t get [a specific chemotherapy]? Well the oncologist said that if the cancer comes back they won’t have anything to give me. And my sister was like “isn’t that the whole point is for the cancer not come back?” So I just felt like they were withholding a higher dose of chemo that would help me in the first run and…these are just personal things. So, I just go into every situation thinking of whether or not I am getting at least the standard level of care or less than that.” “All the people around me are on 40–50 pills. This is too much medicine. They overmedicate people! Then they walk around like zombies, you know, and that is a problem because doctors are the biggest drug dealers I know.” “I’m saying that they’re not telling you everything that you need to know. They’re telling you only what they’re obligated to tell you. So there’s a lot of things they hold back. Just like I’m sure when they get a chart and they look at the chart, the age, the background, the occupation…All this, I’m sure they make some kind of decision of how far they wanna go with this person when compared to this person right here whose net worth might be $300,000 a year. I think that person they might really pay more attention to than this person who is getting public assistance.” “I think research is good. But it depends on the research you’re doing. If you’re gonna talk over my head, what good is the research that you did to benefit me? So for common people like us, we been fooled so many times. We’re tired.” “Black culture is afraid because for years, you know they used us as experiments as you know years ago when they experimented in the early years…and sometimes we still have that fear.” “I am tired of being pitied, you know. If I get a new doctor referral, I’m tired of being pitied on. I’m the same as you. I am not less than you. I am the same. That’s my goal just to be the same, no judgment.”

**Table 2:** Interpersonal factors that influence trust for the healthcare system and/or research: Illustrative examples.

Social Ecological Model Level	Representative Quotes
Interpersonal—formal (medical providers, research staff) and informal (social support systems)	“...Because that’s important to have some cultural appropriateness there...Because there is an increased level of understanding of perspective one is coming from. More knowledge about what really goes on within a particular type of family and lifestyles. A sense of comfort, because likeness kind of breeds some sense of trust. There’s some sensitivity there and a greater depth of understanding.” “I would think that communication…researchers…to build confidence I think that communication is key. I think it’s very important for the doctors and researchers to communicate with the people and make them…have good bedside manners…make them feel that they are concerned and they do wanna help. I think that will bring a good response from people and a willingness for people to want to partake.” “We have to hold the doctors accountable. The doctors give the wrong information so it’s up to you to research the information that they give you to make a sound decision. And if you don’t make that decision then they just pass you on and experiment on you. I don’t want an experiment; I want something that’s gonna help me.” “The doctors or the nurses, anyone who works in any of the hospitals or the office, they need to understand the culture of what deaf people value. We need to educate them on a few things; awareness about how deaf people think, how deaf people do things. Most people need a lot of visuals in the doctor’s office to explain things. If you have no visuals, it’s hard to understand. Many people’s doctors and nurses, they need more education on how to understand our culture, our everyday life and what it’s like.” “I want to add that when you have a family member who knows things before the deaf patient does; that’s a big no. They need to provide interpreters for the deaf person. No one should have to ask their family member to explain what’s going on.”

**Table 3:** Institutional-level factors that influence trust for the healthcare system and/or research: Illustrative examples.

Social Ecological Model Level	Representative Quotes
Institutional—organizational characteristics, including hospital/clinic climate, patient accommodations and supportive resources, financial policies, appointment scheduling, respect for persons	“We do not trust the system because simply the system cannot be trusted. It has moved from what it should be. Healthcare should be first; it became a business. The hospital is no more than a business right now.” “But I know people that go [to hospital] and they tell me that even though they accept high and low income people, you can tell which are getting the high and the low treatment. At [hospital], they accept you but they don’t give you the care that they’ll give somebody else. They don’t want you there. And they let it be known. The care is different.” “It does always seem to come back to money. My problem is the billing process. You can go and have treatment. And then you don’t get a bill for months later. And you don’t know what tests you had that required all these thousands of dollars. You’re not told up front. You don’t even know what all the tests are for. Or what they drew the blood for. It’s like, they just...are they doing this for research or what? But you end paying for it because it’s part of a test that you didn’t know anything about. And the time in which you get the bill, you can’t connect the two. You just get a big bill in your name.” “Since my husband has been diagnosed with cancer, we have spent a lot of time in hospitals and being at different appointments. I think one of my issues is that my trust over this process has shaken a bit. Because the time the time they spend with you is so short. Even when you’re dealing with something that is a chronic illness. You take the time from work and other things that you have going on. They rush you out and they don’t give you enough information and it shakes your trust in the healthcare system. The better your insurance the better care you receive. It has shaken my trust in the healthcare system a little bit.” “When it comes to medical research the trust has been broken a long time ago in the African American community. And I don’t know if the healthcare system has done a great job at repairing that. And some people will say ok and not be aware of it, and I don’t that think that’s good either. I think a person should be informed before they make a decision to be a part of research. I don’t think the healthcare system has done a great job to ease the minds of the African American community around research so that they will feel more comfortable to ask questions and participate. And you want to support the global community doing this research because good can come out of it, but people are scared to do it. I want to help others, but is this going to hurt me or my family in the process? Maybe more information about the benefits around research and the benefits to the individuals could be communicated better.” “They bring me the VRI [video remote interpreter] machine and then I have to say explain it again and again and again. So, most of the time, if I plan to go to the MRI [magnetic resonance imaging], I can’t because I have a Cochlear. But for example, MRI, CAT [computerized axial tomography] scan and any of those types of things, ultrasound. It’s a dark room so you know the VRI can’t see me. So, how can they possibly interpret for me?” “They did not have an interpreter. They just wanted us to read their lips and depending on me to read lips for him. They didn’t understand. They were having conversations, turning every which way and I can’t read their lips. When they are doing that and they’re using big words, big fancy words, that don’t mean anything to me. So, it doesn’t matter if I’m hard of hearing. I still need to have a live person interpreter, not that VRI.

**Table 4:** Community-level factors that influence trust for the healthcare system and/or research: Illustrative examples.

Social Ecological Model Level	Representative Quotes
Community—relationships among institutions and communities (e.g., built environment, neighborhood qualities, housing, businesses, and transportation)	“The community in which we live, we have certain soil that they dump things in around here in the neighborhood. I think that plays a great...it’s a part of what type of cancer...the exposure to certain things is what I’m speaking of. I think that plays a big part of cancer, because they dump recycling here on [name] road. They allow people to put that dump over there and the people were exposed to all the foul odor and all of that stuff, so it could not have been healthy for them breathing this...they couldn’t sit on the front porch.” “Same kind of way with the air. You know, I remember when [company] was there by the lake. You knew the minute you hit [the city] because you smelled all that stuff. Just imagine, you live right there where that plant was, how those people’s lungs are.” “You know it’s the financial when you have like our race of people situated in an area, that dumping, the water, everything is not what would be out in all those upper suburbs.” “If I hear that we live in a food desert one more time, because everyone lives in a food desert, because stores are just not in our neighborhood...all these pundits on television that tell you to shop in our perimeter. That’s just hard to do.” “You keep sharing information about patient navigators, social workers, etc. That’s one of the things that seems to be missing within our community across the board. So there needs to be that type of information readily disseminated, communicated and the community made aware of. So there needs to be some kind of vehicle or linkage when someone goes to the doctor that’s automatically communicated in layman’s terms instead of on page five of the documents of what you’re signing. That’s the kind of thing that would empower a community where things won’t have to go two years or five years where there could be some intervention prior to that so that people don’t get their hopes up about what {name of program] can do. That’s the type of information that needs to be communicated more in layman’s terms.” “So, one of things we should try to get to the communities back at some grassroots-level programs like a Black barbershop outreach program. And aim it at Black men and disseminate information right in the barbershop; that’s a man’s man cave, they go to the barbershop. We got the big clinic and sometimes facilities are too big or overwhelming. There’s a disconnect. We need to make sure we have those grassroots very solid [and] ongoing and sufficiently funded outreach and education programs. Because that’s where some of the empowerment will come from.”

**Table 5:** Policy-relevant factors that influence trust for the healthcare system and/or research: Illustrative examples.

Social Ecological Model Level	Representative Quotes
Policy—local, state, national, guidelines, recommendations, policies, and laws that influence healthcare, research, access to resources, insurance	“It is imperative that you have a primary care [physician] who follows you regularly or that you check in with regularly. Because if you have a primary care physician who is aware of your culture, race, and your family history, it is a lot easier for them to write a justification to get a man screened at 30 [years old] versus older. More than likely they are not going to put their neck on the line to give a prescription or an order for the test you need because you don’t meet that age criteria. Which if you have a primary care and they understand that part about you, it’s a lot easier for them to say to an insurance company I know your screening guidelines are age 50, but this African American man has a family history or his body is reacting this certain way that he needs to bypass the age of 45 for African Americans to 30.” “Doctors have their orders…they are working with pharmaceutical companies there is legislation involved. There are all these different moving parts, you are sometimes a number in there and you have to advocate for yourself and the way you learn to advocate for yourself is through your connections with other people in the community to support you.” “You have to listen to your body. And if something is wrong, you need to tell the doctor ‘look I need you to sit here and take time with me.’ And if you don’t feel right with that doctor, go to another doctor. Sometimes it’s good to go to different doctors. I believe it shouldn’t have to do with your insurance. Nobody should die because they don’t have insurance. Nobody should not get treatment because they don’t have insurance. That’s not right.” “I would like to know why people like me pay for insurance if they got Medicare and I still have to pay a co-pay? And my Medicare went up. What’s the point of having insurance if it has to come out of your pocket?” “And one of the problems was a lot of the medications had side effects that were worse than the conditions that they had. So, we saw a lot of people actually die from the side effects they were getting. And nothing would happen, because nothing was illegal.” “Well, that all depends on my insurance. I can’t pick my own deaf-friendly doctor. I have to go through my insurance first and that limits who I can go to. I have to pick one off the list.” “I wish the insurance would be able to take care of the deaf better.” “I want to know about all the medical schools, their training to become doctors or nurses. Do they have a class to understand access to disabilities? I just want to know that if they a class, anyone to teach them simple things or teach them deep.”

## Supplementary Materials

Supplementary material can be found at https://www.mdpi.com/1660-4601/16/18/3280/s1.
