# Supplementary material for: Understanding Multilevel Factors Related to Urban Community Trust in Healthcare and Research
Source: Int J Environ Res Public Health. 2019 Sep 6;16(18):3280. doi: 10.3390/ijerph16183280 (PMC6765868; doi:10.3390/ijerph16183280)
Supplement: Supplementary file 1 [file ijerph-16-03280-s001.pdf]

CASE WESTERN RESERVE UNIVERSITY | CASE COMPREHENSIVE CANCER CENTER

Office of Cancer Disparities Research

Forward Movement Project

Community Listening Tour

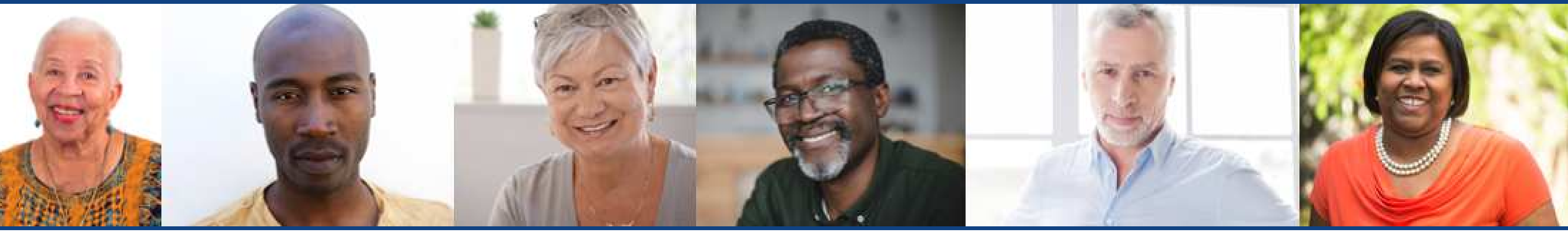

PROJECT OVERVIEW — In early 2018, greater Cleveland residents, in nine neighborhoods, engaged in "town-hall" discussions about cancer risks, community trust in healthcare, and research. The goal was to learn about patient experiences.

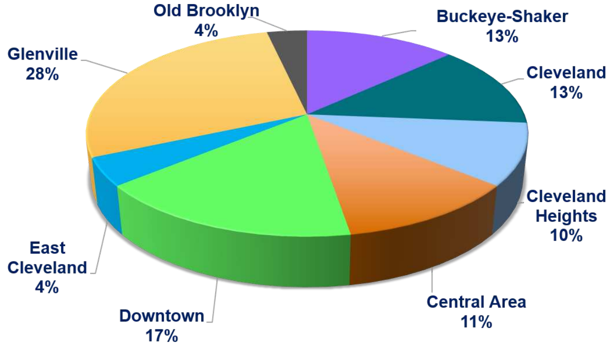

130 PARTICIPANTS

RACE

Black	80%
White	17%
Other	3%

SEX

Female	68%
Male	32%

HEALTH INSURANCE

Yes	97%
No	3%

CHRONIC HEALTH CONDITION

Yes	71%
No	29%

Community Voices

1 Trust — Here's What You Shared:

- Overall, trust in the healthcare system is low because of current and past experiences
- Trust is lowered when doctors and nurses don't seem to care about every patient
- Healthcare is seen as big business — making money is most important
- Patients are treated differently based on their insurance and where they live
- Doctors and drug companies are working together to push medications

2 Healthcare Experiences — Here's What You Shared:

- Overall, most patients had negative experiences with the healthcare systems (e.g., feeling rushed, poor communication, no empathy)
- Patients with good experiences had doctors who spent more time with them
- Deaf and hearing impaired patients prefer live interpreters, and often feel disrespected
- High medical bills arrive months after the visit — out of pocket costs are unknown and insurance policies are not clear

3 Research — Here's What You Shared:

- Research is important and needed to test new treatments (e.g., drugs)
- Trust for medical research was broken in the African American community years ago and hasn't been repaired
- There are concerns about joining research clinical trials testing new medications
- Results of studies are not shared with the community; so, they are not helpful

Office of Cancer Disparities Research
Case Comprehensive Cancer Center
Monica Webb Hooper, PhD
216.368.1122
caseccc-ocdr@case.edu

©2018 All rights reserved.

CASE WESTERN RESERVE UNIVERSITY | CASE COMPREHENSIVE CANCER CENTER

Office of Cancer Disparities Research

Forward Movement Project

Community Listening Tour

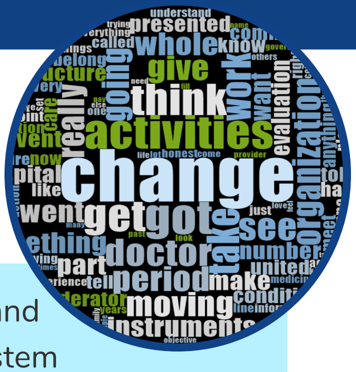

"The Listening Tour was an amazing opportunity to appreciate the wisdom of our diverse community members. The Case Comprehensive Cancer Center will use this information to forward our mission to reduce cancer disparities and death in our community."

- Stanton L. Gerson, MD
Director, Case Comprehensive Cancer Center

PROJECT RESULTS: There are perceived racial disparities in healthcare and distrust for medical research. Patients desire change in the healthcare system and how researchers connect with members of the community.

Ideas For Change — We Used What We Learned From You:

Recommendations for healthcare leaders and researchers — Ideas for change include:

- Train hospital staff to treat patients with care, warmth, and concern
- Share programs and services with all patients (e.g., music therapy, stress management, or tobacco treatment)
- Train hospital staff to make sure patients understand the information given to them
- Make sure the right services are available for patients with disabilities (e.g., live interpreters)
- Get community input before and after research — share results with patients and the larger community

You Are Your Best Health Advocate — Here Are Some Questions To Ask:

At Your Doctor's Visit

- What can I do to stay healthy or improve my health?
- Is this the best treatment option — would you recommend this to your family?
- Are there any programs or services to help me (e.g., patient navigation, financial counseling, spiritual care, or social work)?

About Research Participation

- What is the goal of this research study?
- How will this study help me?
- What are the risks?
- How can I be sure I am protected?
- How will the results be shared with the community?

We Heard You & Thank you!

"This experience allowed me to realize the nuances in healthcare access problems among community members which must be taken into account for a better understanding of factors contributing to unequal cancer burdens in respective ethnic groups."

-Dr. Kishore Guda
Case Comprehensive Cancer Center

"This experience has been extremely valuable to me. It was good not only to hear confirmations on some things that I thought were important to the community, but also to hear some new insights. I plan to incorporate these into my research moving forward."

-Dr. Cheryl Thompson
Case Comprehensive Cancer Center
